# Salmagundi

**Published:** 1886-04

**Authors:** 


### SALMAGUNDI.

EDITED BY E. G. BETTY, D.D.S.

M. PASTEUR has been decorated by the King of Italy.

---

THE credit of the ground-hog as a weather prophet, stands high this spring.

---

AN Augusta (Maine,) lady has ordered a set of false teeth for her old pony.

---

NEW LONDON, CONN., claims to have a man sixty-five years old who has never had a tooth.

---

"THE three-cent nickel is not a highly valuable coin, but on the contribution plate it makes just as good an appearence as a dime."

---

WHY is the man who plays the big fiddle, the most lucky man in the orchestra? Because he has a standing job.

N. B.—This is original.

" A CASE of death from cocaine applied to relieve the pain of a decayed tooth is reported by Prof. R. Ogden Doremus to the New York Medico-Legal Society."

---

THE people of this country use annually about 150,000,000 steel pens. Of these probably fifty (?) are wielded in the service of dentistry! Don't all contribute at once.

---

URETHAN, an æthyl ether of carbamin acid, is a new hypnotic that is said to have a very prompt effect. It is administered in doses of eight (8) grains taken from one to three times at night before retiring.

---

A LITTLE boy of this city, about five years old, announced his ability the other night to frame his own prayer, and proceeded: " O, Lord, make me a good boy, and if at first you don't succeed, try, try again.'--*Hartford Courant.*

---

SIR ANDREW CLARKE defines health as " that state in which the body is not consciously present to us, the state in which work is easy and duty not over great a trial, the state in which it is a joy to see, to think, to feel, and to be."

---

IT is said that "a chemist has distilled an extract from coal-tar, two hundred and thirty times sweeter than sugar." Dentistry will boom when that extract becomes an article of daily use—the little bugs will then not have to make their own sugar and lactic acid you know.

---

THE daylight mornings come at length, the gladdest of the year, when buckwheat cakes and sausage balls, at breakfast don't appear, but in their stead are biscuits hot, besmeared with maple sweets, and fresh-smoked ham and new-laid eggs delight the man who eats.—*Daily Paper.*

THE Chicago College of Dental Surgery announces that "*there is no fee for graduation,*" immediately under the statement in the fee list: "Final Examination Fee—Fee not returnable, $25." This is very much like a grey horse of another color, or a distinction without a difference.

---

"MY DEAR," said a Sommerville mother, annoyed at some incautious remarks of her little girl, "why can't you keep a secret?"

"Because," said the little mischief demurely, "two of my front teeth are gone, mamma."—*Sommerville Journal.*

---

OPPLUS' method of concealing the smell of Iodoform "consists of mixing one part of freshly ground coffee with two parts of Iodoform. The result is most satisfactory, it being difficult to detect the presence of the Iodoform, and the effect lasts for a very considerable portion of the time, *i. e.*, three weeks or a month. The coffee is also accredited with antiseptic qualities."

---

THE following clipping from a daily paper may not be uninteresting as another case of death from strangulation produced by the lodgment of a plate in the throat, though not so interesting as those reported by Dr. Jay at the *M. V. D. A.*, last month:

XENIA, O., March 17.—A singular accident, which resulted in the death of David Strong, aged sixty-eight years, occurred at Medway, a small village near the Clarke and Greene County line, yesterday at noon. Mr. Strong was engaged in eating his dinner, when he suddenly commenced to choke and grow black in the face. The members of his family at once went to his rescue, and tried to relieve him, but were unable to do so, and a messenger was dispatched for a physician. Before medical assistance arrived, Strong was a corpse, death having resulted from strangulation. An examination by the physician showed that he had partially swallowed the upper row of his false teeth while engaged in eating his dinner, and that they had lodged in his throat in such a manner that he was unable to dislodge them, or to make the members of the family understand what was the matter.

---

ECHOES FROM THE M. V. D. A.

DR. BERRY'S dog story was enjoyed by all, and it takes Dr. B. to tell a story.

DR. ROSE'S maiden effort was well received; it is printed elsewhere in this issue of THE REGISTER.

DR. WATT was on hand looking better than we had expected; so much for the principle of "git thar."

DRS. FRANK HUNTER, C. M. Wright and N. S. Hoff, will make a "rattling good" Executive Committee.

The forty-second was one of the best meetings for years past, the reading of the papers, discussions and proceedings occupying but two days, so systematically did the work go on.

DR. W. S. HOW, of Philadelphia, made a most interesting exhibition of instruments for forming gold crowns with certainty and speed. He also showed specimens of simple and effective bridge-work.

DR. REHWINKEL exhibited a number of specimens of Herbst's Method, (made by that gentleman himself,) and gave a good lecture upon the subject, to the pleasure of many who had never before had an opportunity of seeing the process.

DR. J. S. CASSIDAY raised a point, which if elaborated on paper, would make mighty interesting reading, and we hope he will take the hint and send it to THE REGISTER. We refer to his statement that a germicide may be neither antiseptic nor disinfectant, and that all antiseptics and disinfectants are by no means germicides.

DR. G. W. KEELY is too modest by half. He had made a kind of promise to exhibit some cases of irregularities, illustrating them with models and paintings, and we had looked forward to the treat with impatience as we have become a disciple on the irregularity question. We shall have him next year without fail, and give him all the time he can use.

DR. H. J. MCKELLOPS, of St. Louis, came with his pockets so full of "little tricks" that he reminded us of Dr. Baxter, of Vevay, Ind. One of the tricks was simply a little bit of spunk tied in the center of a strand of silk thread, to be moistened in sandarac and drawn between the necks of the teeth, and so left that it will force the rubber dam high up above the margin of the gum. He also showed a set of compound matrices, some of steel and some of bronze, the invention of Dr. Miller, of Altoona, Pa. They need to be seen to be appreciated. One of the neatest is a pair of pliers with one beak concave on the end the other convex, made for the purpose of depressing or elevating the cusps on gold crowns. The doctor exhibited other instruments, all of which were interesting to hear about.

